# Removal of cement-augmented screws in distal femoral fractures and the effect of retained screws and cement on total knee arthroplasty: a biomechanical investigation

**DOI:** 10.1186/s10195-021-00568-w

**Published:** 2021-02-27

**Authors:** Dirk Wähnert, Niklas Grüneweller, Boyko Gueorguiev, Thomas Vordemvenne, Dominic Gehweiler

**Affiliations:** 1 Department of Trauma and Orthopedic Surgery, Protestant Hospital of Bethel Foundation, University Hospital OWL of Bielefeld University, Campus Bielefeld-Bethel, Burgsteig 13, 33617 Bielefeld, Germany; 2 AO Research Institute Davos, Clavadelerstrasse 8, 7270 Davos, Switzerland

**Keywords:** Screw augmentation, Distal femoral fracture, Biomechanics, Total knee arthroplasty, Osteoporosis

## Abstract

**Background:**

Given the increasing number of osteoporotic fractures of the distal femur, screw augmentation with bone cement is an option to enhance implant anchorage. However, in implant removal or revision surgeries, the cement cannot be removed from the distal femur without an extended surgical procedure. Therefore, the aims of this study were to investigate (1) whether cement augmentation has any influence on screw removal and removal torque, and (2) whether the implantation of a femoral component of a knee arthroplasty and its initial interface stability are affected by the remaining screws/cement.

**Material and methods:**

Eight pairs of fresh-frozen human female cadaveric distal femurs (mean age, 86 years) with a simulated AO/OTA 33 A3 fracture were randomized in paired fashion to two groups and fixed with a distal femoral locking plate using cannulated perforated locking screws. Screw augmentation with bone cement was performed in one of the groups, while the other group received no screw augmentation. Following biomechanical testing until failure (results published separately), the screws were removed and the removal torque was measured. A femoral component of a knee arthroplasty was then implanted, and pull-out tests were performed after cement curing. Interference from broken screws/cement was assessed, and the maximum pull-out force was measured.

**Results:**

The mean screw removal torque was not significantly different between the augmented (4.9 Nm, SD 0.9) and nonaugmented (4.6 Nm, SD 1.3, *p* = 0.65) screw groups. However, there were significantly more broken screws in in the augmented screw group (17 versus 9; *p* < 0.001). There was no significant difference in the pull-out force of the femoral component between the augmented (2625 N, SD 603) and nonaugmented (2653 N, SD 542, *p* = 0.94) screw groups.

**Conclusion:**

The screw removal torque during implant removal surgery does not significantly differ between augmented and nonaugmented screws. In the augmented screw group, significantly more screws failed. To overcome this, the use of solid screws in holes B, C, and G can be considered. Additionally, it is possible to implant a femoral component for knee arthroplasty that retains the initial anchorage and does not suffer from interference with broken screws and/or residual cement.

**Level of Evidence:**

5

## Introduction

Only 6% of all femoral fractures occur at the distal femur, so they are relatively rare [1]. However, approximately 50% of all distal femoral fractures occur in elderly patients, implying that the number of osteoporotic fractures is rising. In 2010, 2.46 million new fractures due to osteoporosis were reported in the five largest countries of the European Union (France, Germany, Italy, Spain, UK) and Sweden [2].

Supracondylar fractures are the most common type in the elderly due to attenuated bone quality, especially within the metaphyseal part of the distal femur [3, 4]. The osteosynthesis of such fractures is challenging due to reduced implant anchoring [5, 6]. Indeed, complication rates reported in the literature range from 18 to 35% following locked plating [7, 8].

**Fig. 1:**
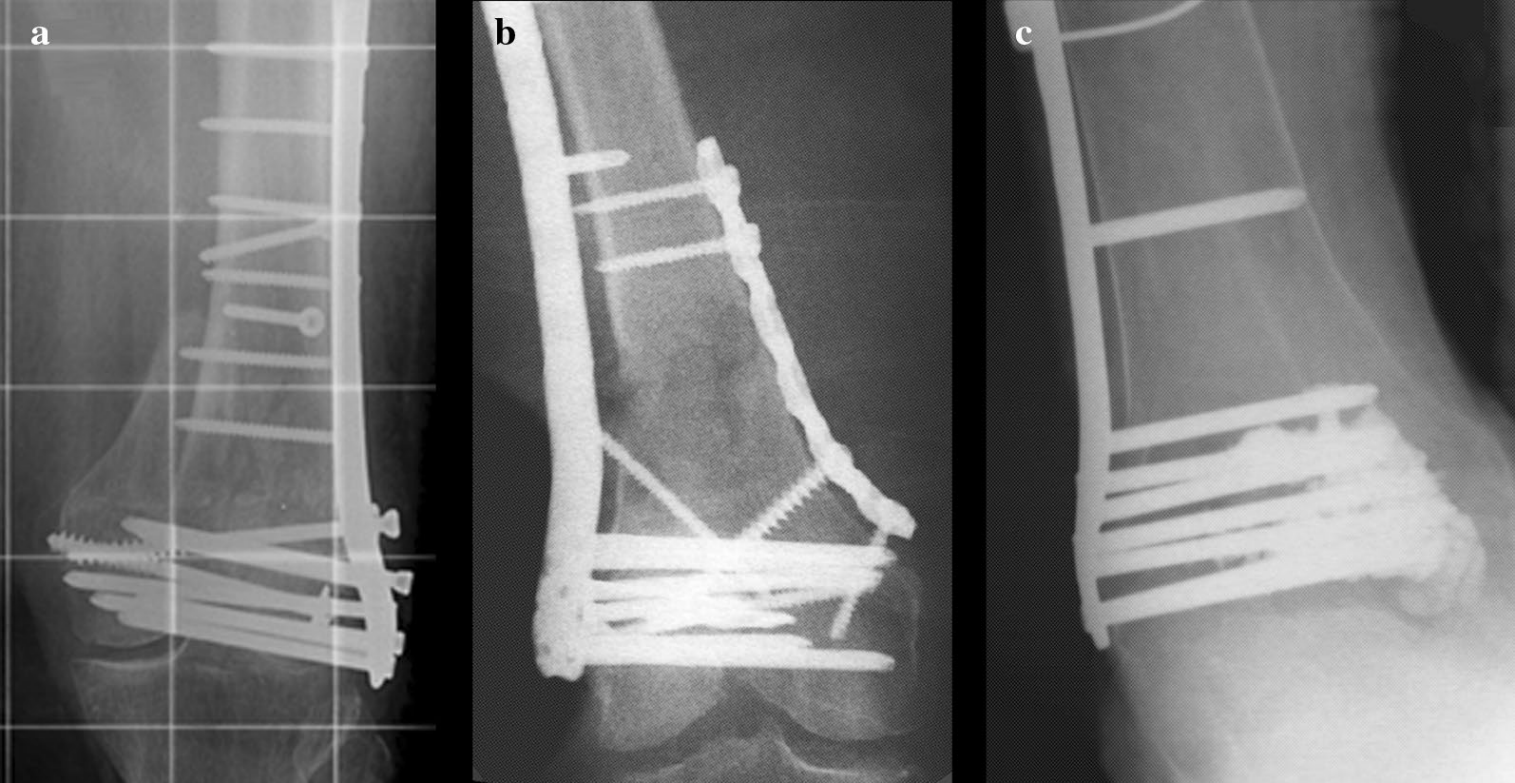
Distal femoral fracture cases. **a** X-ray of a distal femoral fracture after locked plating in anteroposterior projection; construct failure due to screw loosening and secondary loss of reduction is apparent. **b** X-ray of a distal femoral fracture treated with double plating to increase construct stability. **c** X-ray of a severe osteoporotic distal femoral fracture with locked plating and screw augmentation to enhance implant anchorage and construct stability

Figure 1 shows an example of a construct failure after locked plating of an osteoporotic distal femoral fracture (Fig. 1a), as well as two options to enhance its construct stability (Fig. 1b, c). One of the options considers insertion of a second plate on the medial side, which results in considerable soft tissue damage and significantly increased surgery time. The other option – called implant augmentation – aims at enhancing implant anchorage in osteoporotic bone by using bone cement to encase screw tips and increase construct stability. In previous biomechanical investigations, our group was able to show the benefits of screw augmentation at the treatment of osteoporotic distal femoral fractures [9, 10]. However, due to the use of nondegradable polymethylmethacrylate (PMMA) based bone cements for augmentation, removal during revision surgery is impossible without massive bony destruction.

Therefore, the aims of this study were to investigate whether (1) screw removal is possible following augmentation, (2) the screw removal torque is affected by the augmentation, and (3) there is interface instability in cases when knee arthroplasty is required after screw augmentation.

We hypothesized that augmentation significantly affects (1) the screw removal and (2) the interface stability.

## Material and methods

All human specimens in this study were prepared and used in accordance with the “Gesetz über das Leichen-, Bestattungs- und Friedhofswesen (Bestattungsgesetz) vom 04.02.2005, Abschnitt II, § 9 (Leichenöffnung, anatomisch),” which allows donor bodies to be dissected for scientific and/or educational purposes.

### Specimens

Eight pairs of fresh-frozen human female cadaveric distal femora aged 86 years on average (range 81–92 years) were used in this study.

### Bone mineral density

Bone mineral density (BMD) was measured by means of high-resolution peripheral quantitative computed tomography (HR-pQCT, XtremeCT, Scanco Medical AG, Bassersdorf, Switzerland) operated at 60 kVp and 900 µA, with 750 projections, an acquisition time of 200 ms, and a resolution of 123 µm. Following an established routine, BMD was evaluated within a cancellous region of interest. Selection was based on the largest condyle diameter with a thickness of 180 slices. A semiautomated segmentation procedure for the cancellous bone was applied [11].

### Instrumentation, augmentation, and biomechanical testing

All specimens underwent an investigation beforehand to check the feasibility of cement augmentation for enhancement of implant anchorage in osteoporotic distal femoral fractures [9].

In brief, the distal 7 cm of each femur was cut parallel to the joint line and plated in a simulated AO/OTA 33 A3 fracture model using a locking compression plate for the distal femur (LCP DF, DePuy Synthes, Zuchwil, Switzerland) made of titanium alloy. Distal fixation was performed with monocortically placed (at a distance of 2–5 mm to the medial cortex), cannulated and perforated 5 mm self-tapping locking screws (DePuy Synthes) tightened with a 4 Nm torque limiter.

The specimens from each pair were then randomly split into two study groups with equal numbers of right and left distal femora. No screw augmentation was performed in group I. One milliliter of PMMA-based bone cement (Traumacem V+; DePuy Synthes) was injected through each of the distal screws of the specimens in group II. Cement curing was enhanced by storing all the augmented specimens for 6 h at room temperature.

Biomechanical testing was performed on a servohydraulic testing machine (MTS 858 Mini Bionix II; MTS, Eden Prairie, MN, USA) equipped with a 4 kN load cell. All specimens underwent progressively increasing cyclic sinusoidal axial loading at 2 Hz until construct failure [9]. Whereas the valley load of each cycle was kept at a constant level of 100 N, the peak load increased at a rate of 0.05 N/cycle, starting from 750 N. 

### Peak torque for screw removal

**Fig. 2:**
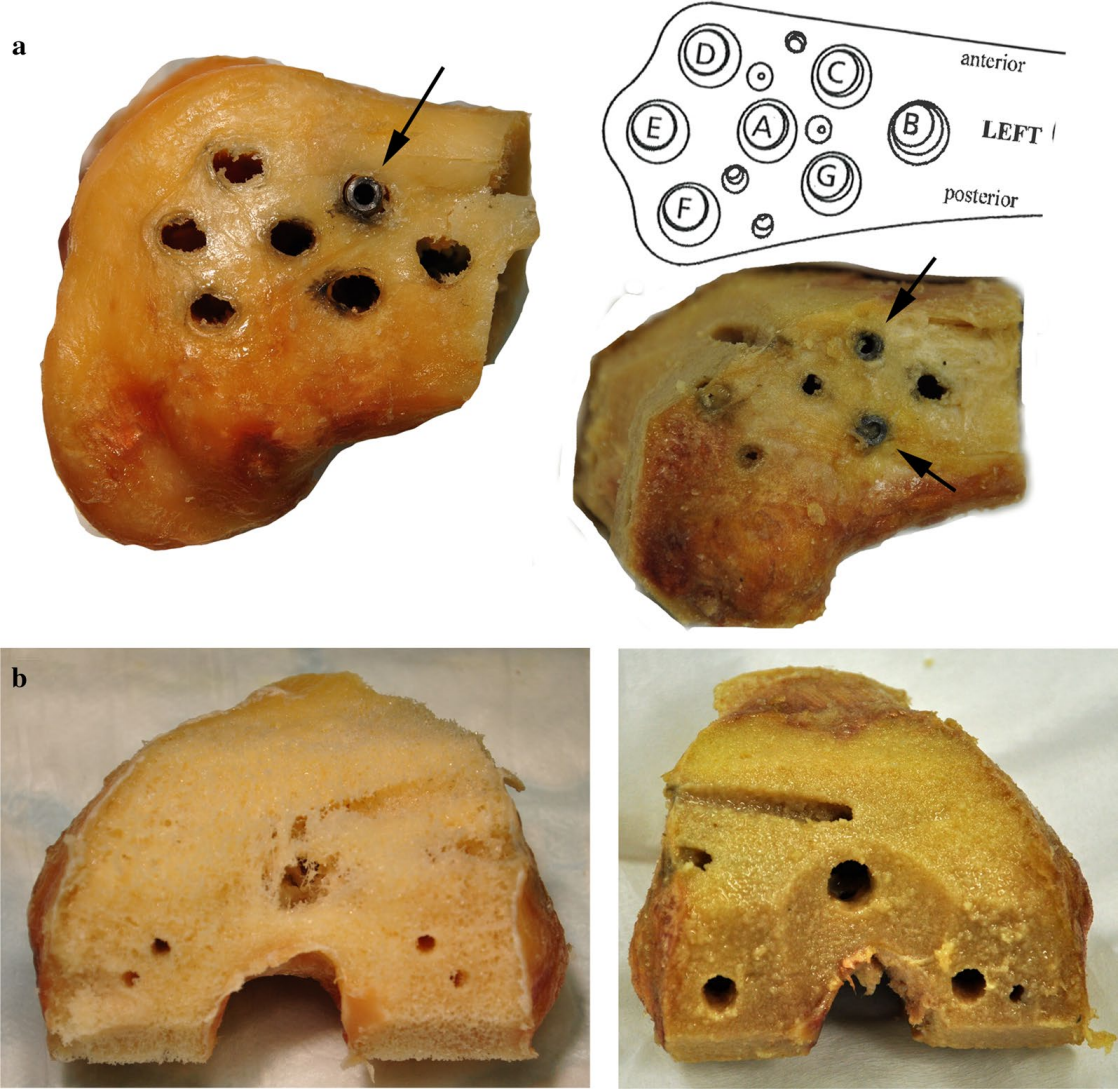
Knee arthroplasty instrumentation. **a** Images of two distal femoral condyles after implant removal; broken screws are present in holes C and holes C and G, respectively (indicated by* arrows*). Broken screws were principally detected in holes B, C, and G (see schematic). **b** Images visualizing situations after completing all resections. No interference with the remaining screws or cement was observed

Photo and X-ray documentation of the construct failure modes was performed after biomechanical testing. The failure mode significantly differed between the groups; screw cut-out was observed in all nonaugmented specimens, whereas screw and plate breakage occurred in all augmented specimens [9]. Following, implant removal was performed with measurement of the peak torque using a torque measuring device (Mecmesin, Slinfold, UK) attached to a hand screwdriver. All broken screws were left in the femur to avoid permanent bone damage and to investigate the potential interference of those screws with the resection for the following knee arthroplasty (Fig. 2a).

### Knee arthroplasty instrumentation

**Fig. 3:**
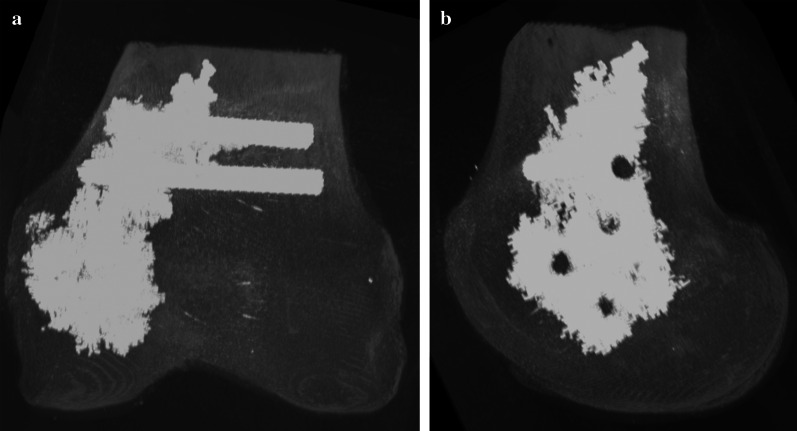
Radiographical reconstruction of an augmented specimen after implant removal and before knee arthroplasty implantation: **a** Anteroposterior view; **b** lateral view

**Fig. 4:**
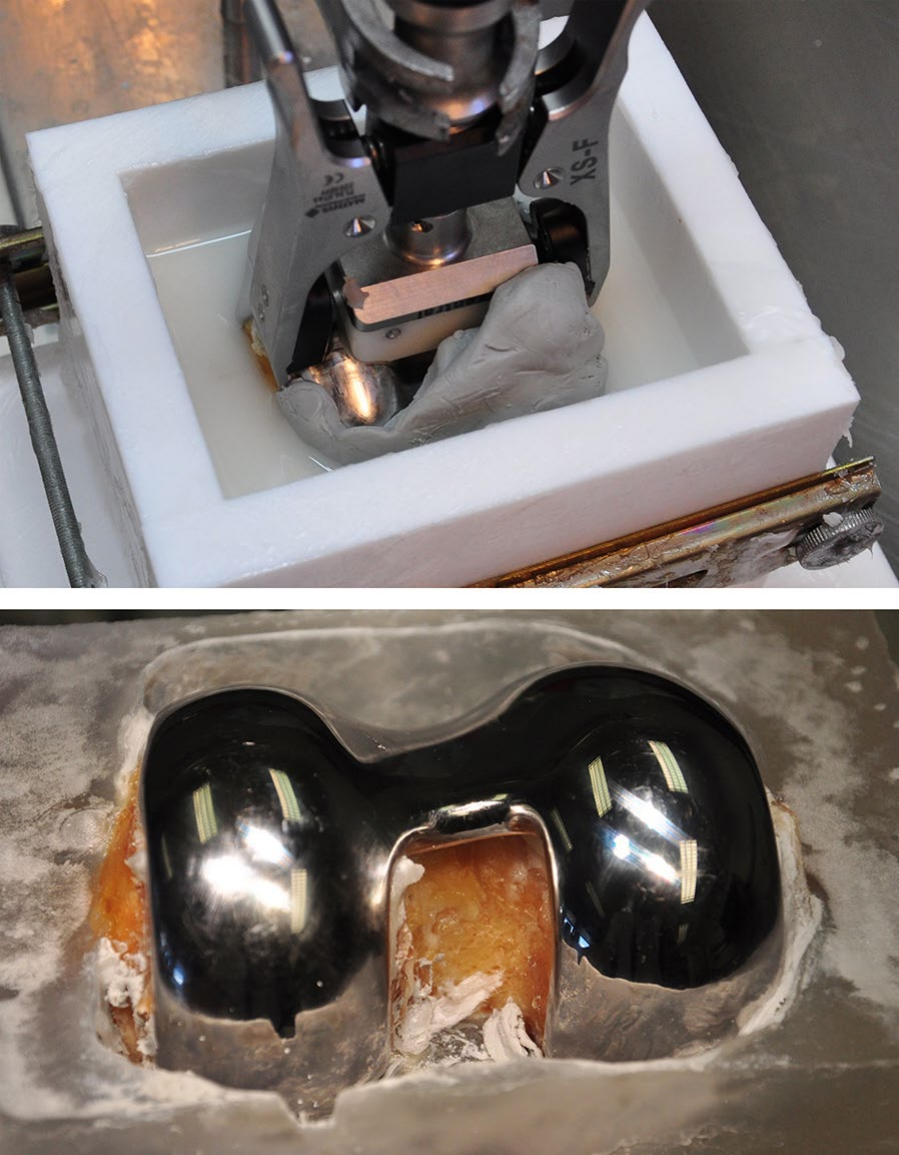
Specimen preparation for pull-out testing. PMMA embedding of the femoral bone. Particular attention was paid on keeping the femoral component free of PMMA by using plasticine (*top*). Situation after plasticine removal (*bottom*)

Following implant removal, the HR-pQCT scanning of each specimen was repeated to check the bony integrity of the condyles (Fig. 3). A balanSys BICONDYLAR femoral shield (Mathys AG Bettlach, Bettlach, Switzerland) was implanted according to the instructions and with the instruments provided by the manufacturer. First, the medullary canal was opened using a balanSys drill. The femoral intramedullary rod was then inserted and the guiding jig for the distal femoral resection was attached. No intramedullary guide insertion problems, related to the remaining screws and cement, were encountered. After aligning the distal femoral cutting block, the resection was performed using an oscillating saw. When all devices had been removed, the rotation and size of the femoral component were determined and the 4-in-1 cutting block was mounted to the distal femur. After checking the correct position of the cutting block, anterior resection was performed, followed by posterior and oblique cuts (Fig. 2b). Following the preparation of the trochlea, the femoral component was prepared with bone cement (C-ment 1, Leader Biomedical, Amsterdam, Netherlands), according to the manufacturer’s instructions. The femoral component was attached to the distal femur via slight hammer strikes. Prior to biomechanical testing, all specimens were stored for 6 h at room temperature for cement curing. The specimens were kept moist with wipes soaked in saline. Thereafter, the specimens were embedded in polymethylmethacrylate (Beracryl, W. Troller Kunststoffe AG, Jegenstorf, Switzerland). Particular attention was paid on keeping the femoral component free of PMMA using plasticine (Fig. 4). Due to the biomechanical testing performed beforehand and the proximal embedding of the specimens, the simulated conditions were comparable to those of a healed supracondylar fracture with implant removal and a metaphyseal defect in the nonaugmented specimens.

**Fig. 5:**
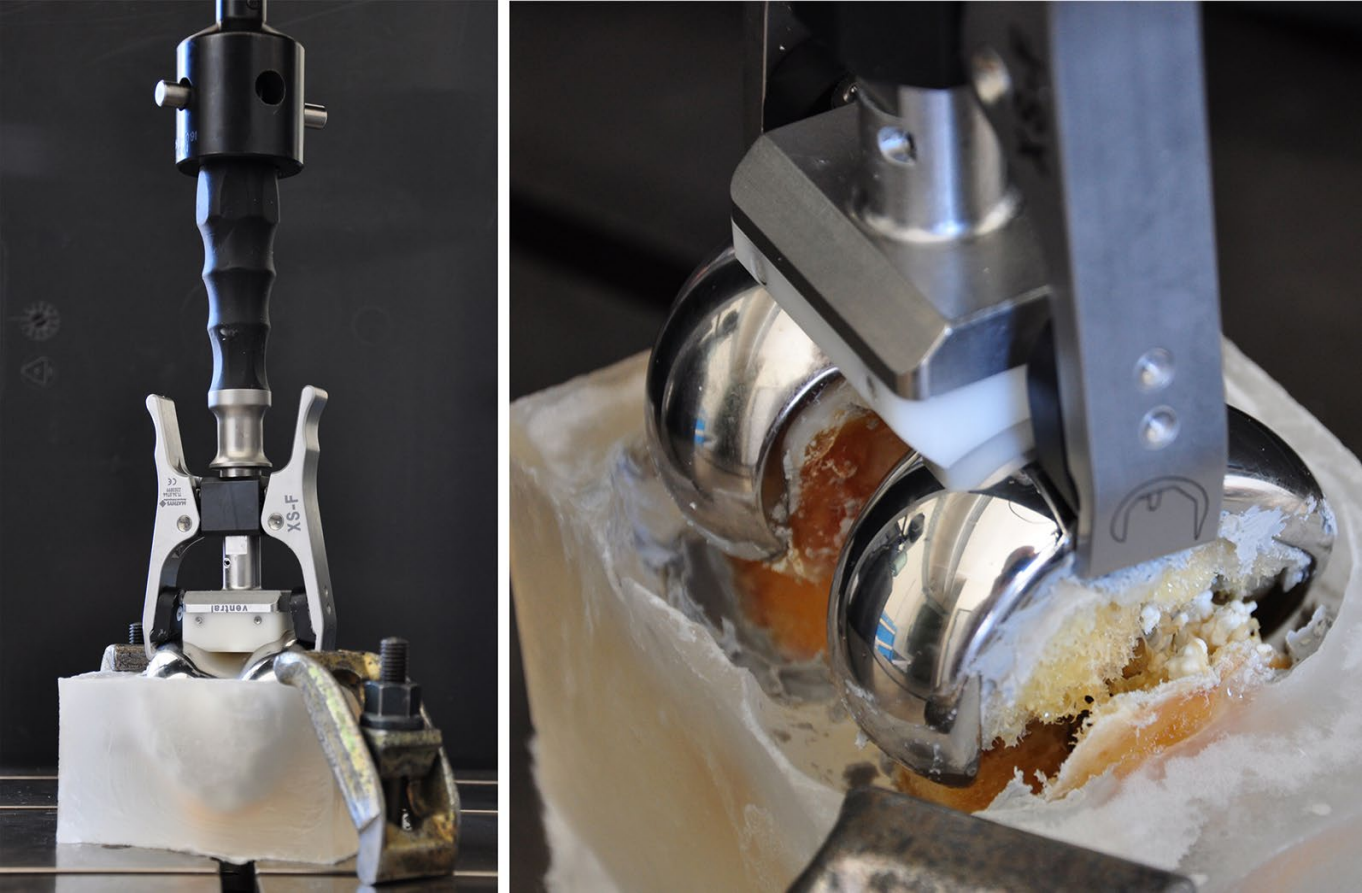
Visualization of the setup with a specimen mounted for pull-out testing (*left*) and a detailed view of the femoral component clamping (*right*)

**Fig. 6:**
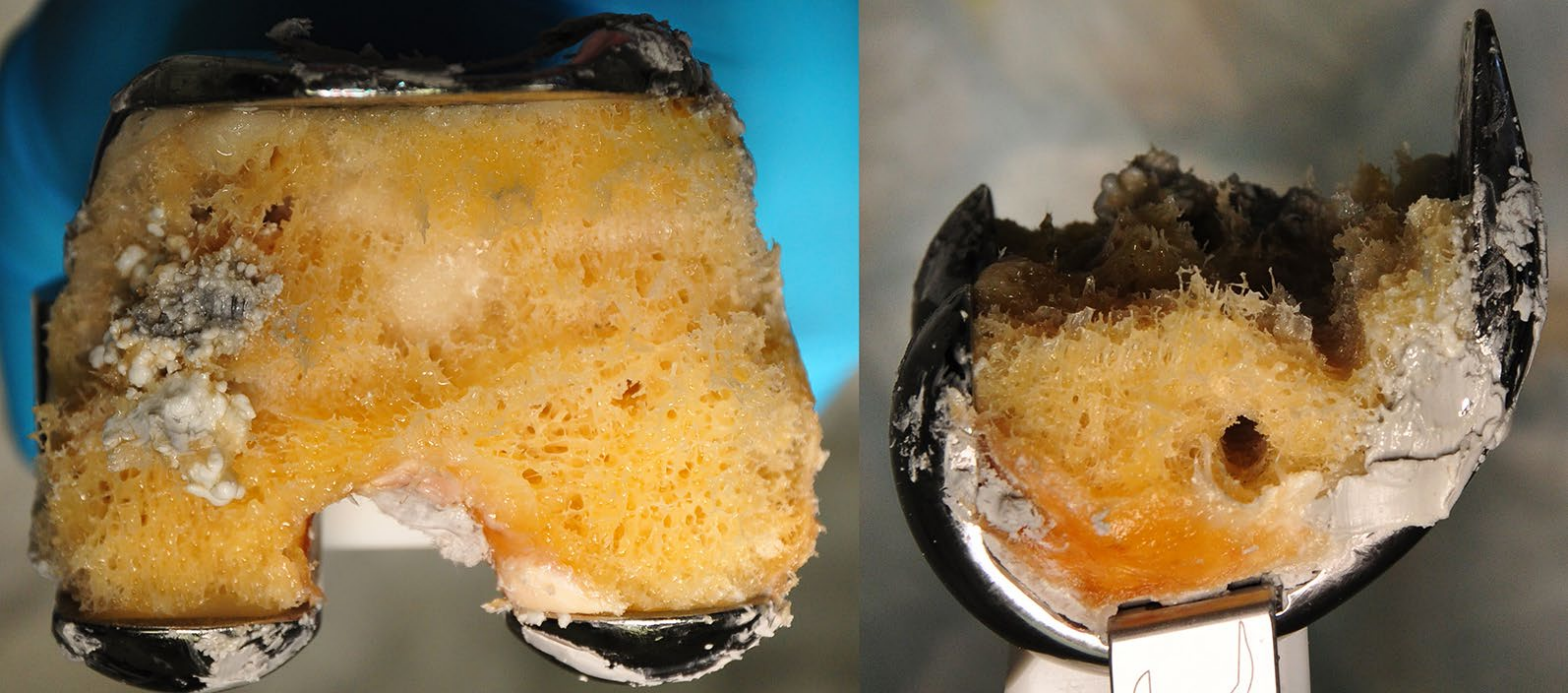
Specimen failure following pull-out testing. Example of the failure mode in the metaphyseal part of the distal femur with the femoral component still connected to the most distal part of the bone

### Pull-out testing

Pull-out testing of the femoral component was performed on an electromechanical testing machine (INSTRON 4302; Instron GmbH, Pfungstadt, Germany) with a 10 kN load cell. Each specimen was fixed to the machine base by clamping its PMMA block (Fig. 5, left). The femoral component was connected to the machine actuator and the load cell using an extractor device (Fig. 5, right). A quasi-static pull-out test was run in displacement control at a crosshead speed of 5 mm/min.

### Statistical analysis

Statistical analysis was performed using SPSS software package (version 24, SPSS, Chicago, IL, USA). The Shapiro–Wilk test was applied to screen and prove the normality of the data distribution related to the bone quality and biomechanical testing parameters. The paired-samples *t* test was used to check for significant differences between the study groups. Fisher’s exact test was applied to detect significant differences between the groups with regard to the numbers of broken screws. Pearson correlation analysis was performed to screen the correlation between the pull-out force and BMD. The significance level was set at *p* = 0.05 for all statistical tests.

## Results

A summary of the results is presented in Table 1.

**Table 1:** Results for the parameters of interest in the nonaugmented and augmented screw groups

	Nonaugmented screw group	Augmented screw group	*p *value
BMD, mgHA/cm^3^			
Mean (SD)	148 (42)	161 (31)	0.13^#^
Median (range)	156 (76–192)	158 (112–201)	
Number of broken screws	9 of 24	17 of 24	< 0.001^*^
Screw removal torque, Nm			
Mean (SD)	4.86 (1.0)	4.97 (0.8)	0.65^#^
Median (range)	4.9 (2.6–7.1)	5 (3.1–6.7)	
Femoral component pull-out force, N			
Mean (SD)	2653 (542)	2625 (603)	0.94^#^
Median (range)	2573 (1878–3860)	2595 (1680–3501)	

*p *value based on ^#^the paired-samples *t* test or ^*^ Fisher’s exact test

### Screw removal

All broken screws were located in the three most proximal plate holes near the fracture line. Nine of the 24 proximal screws were broken in the nonaugmented screw group, versus 17 in the augmented screw group (*p* < 0.001). All screws broke directly at the transition from the screw head to the screw shaft, i.e., directly behind the plate. In the augmented screw group, 7 broken screws were located in hole B (the nearest to the fracture line), 6 broken screws were in hole C, and 4 broken screws were in hole G. In the nonaugmented screw group, 5 broken screws were located in hole G, 3 broken screws were in hole C, and 1 broken screw was in hole B.

The average peak torque for screw removal was 4.97 Nm (standard deviation (SD) 0.8 Nm) in the augmented screw group and 4.86 Nm (SD 1.0 Nm) in the nonaugmented screw group; the difference between the groups was not significantly different (*p* = 0.65).

### Femoral component pull-out

The average pull-out force was 2625 N (SD 603 N) in the augmented screw group and 2653 N (SD 542 N) in the nonaugmented screw group; the difference between the groups was not significant (*p* = 0.94). There was also no significant correlation between the pull-out force and BMD (*p* = 0.15).

### Failure mode: pull-out

In all specimens, failure occurred in the metaphyseal area of the distal femur; the femoral component was torn out with a bony part. The tear ran through the drill channels of the screw holes (Fig. 6).

## Discussion

The present study investigated the biomechanical effects of screw augmentation on revision procedures such as implant removal and knee arthroplasty at the distal femur. During implant removal, we noted that there were significantly more broken screws in the augmented versus the nonaugmented screw group (17 versus 9 out of 24 proximal screws). In both groups, all broken screws were located close to the fracture gap. In addition, all screws broke at the head–shaft transition. Hence, it is assumed that the cannulated screws were overstrained at this position due to plate bending. A possible option would be to use standard noncannulated screws at these vulnerable positions. In our study, there was no significant difference between the augmented and nonaugmented screw groups in terms of average maximum screw removal torque. This finding agrees with previous investigations of solid screws that reported no significant difference between augmented and nonaugmented pedicle screws in relation to their average maximum torque during screw removal [12, 13]. In contrast, several studies concluded that the average maximum torque applied to remove augmented cannulated and perforated screws was significantly higher, reaching values 1.5–12 times higher than those during the removal of nonaugmented screws [12, 14, 15]. To address this problem, the number of screw perforations and their diameter were reduced in the current study to 4 perforations per screw with a diameter of 1.1 mm. Therefore, augmented screw removal was possible without significant increase in torque. All screws were easily loosened by shearing off the cement cloud.

Another concern is the interference with remaining screws and cement during revision surgeries with implantation of femoral components of knee prostheses. We could not find any evidence of interference with the remaining screws and cement in this investigation. Since only the proximal screws were broken, the implantation of a normal bicondylar femoral component could be performed without removing the broken screws. The cement clouds also did not interfere with the osteotomies preformed for femoral component implantation. Previous studies have demonstrated that the use of small cement volumes (e.g., 1 ml per screw or 3 ml per blade) leads to significantly improved implant anchorage [9, 10, 16]. It can be concluded that implantation of the femoral component after augmented plate osteosynthesis at the distal femur can be performed using standard procedures. We also found no significant difference between the augmented and nonaugmented screw groups in terms of initial anchoring of the femoral component. In summary, knee arthroplasty as a revision surgery following augmented plate osteosynthesis appears to be unproblematic.

Nevertheless, there is a scenario for revision where augmented plate osteosynthesis does appear to be problematic, namely in case of infection in the region of the distal femur. In such a case, it is not easy to completely remove both the implant and cement. That is why we consider augmented plate osteosynthesis in the region of the distal femur to be performed only as a rescue procedure, providing the surgeon with a treatment option in difficult cases.

This study has some limitations inherent to all investigations that use a small number of cadaveric specimens. Because of the limited availability of osteoporotic human cadaveric bones, and for ethical reasons, we tried to keep the number of specimens as low as possible. The selected fracture type—supracondylar fracture—represents just one type of clinically occurring distal femoral fracture. However, supracondylar femoral fracture is the predominant form of osteoporotic fracture at the distal femur. In a study of 283 osteoporotic distal femoral fractures by Myers et al., 76% were supracondylar fractures (5% simple and 19% complex intraarticular) [4]. Additionally, this was an in vitro biomechanical study with a pull-out test that did not represent the clinical mode of failure after knee arthroplasty. We performed the pull-out test to investigate any effects of the cement remaining from screw augmentation. No statement can be made about the long-term stability based on the results of this study. We only examined one femoral component from one manufacturer, so it is not possible to draw firm conclusions about other models and manufacturers due to possible differences in design and surgical technique.

## Conclusions

The implant removal torque does not significantly differ between augmented and nonaugmented screws. Significantly more screws failed by breakage in the augmented screw group. Solid screws could be used in holes B, C, and G of the locked distal femoral plate to overcome this issue. Additionally, it is possible to implant a femoral component for knee arthroplasty while retaining the initial anchorage and without interference with broken screws and/or residual cement. However, in case of infection, a complicated surgical procedure would be needed to remove the cement.

## Data Availability

The datasets used and/or analyzed during the current study are available from the corresponding author upon reasonable request.

## Abbreviations

PMMA: Polymethylmethacrylate
BMD: Bone mineral density
HR-pQCT: High-resolution peripheral quantitative computed tomography
